# Supplementary material for: The structural basis for CD36 binding by the malaria parasite
Source: Nat Commun. 2016 Sep 26;7:12837. doi: 10.1038/ncomms12837 (PMC5052687; doi:10.1038/ncomms12837)
Supplement: Supplementary Information — Supplementary Figures 1-10 and Supplementary Tables 1-3. [file ncomms12837-s1.pdf]

Supplementary Information

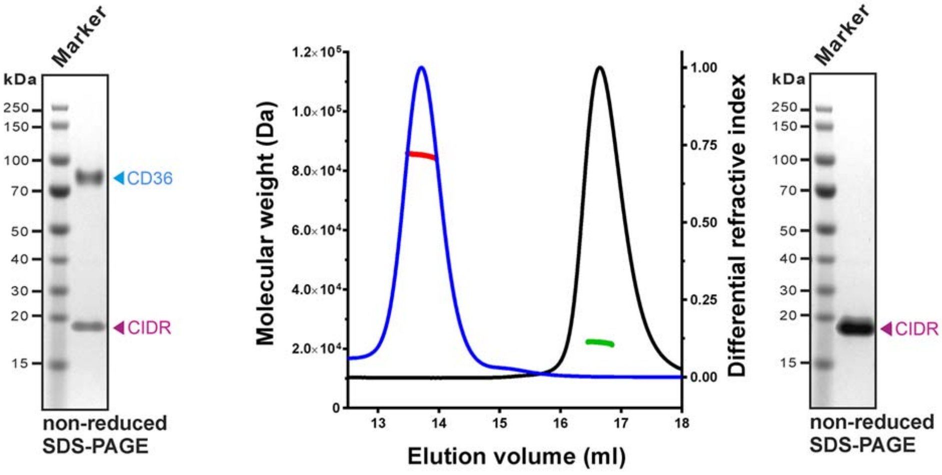

Supplementary Figure 1: Biophysical analysis of the CD36:CIDRα2.8 complex

Analysis of the formation of a 1:1 complex of CD36 bound to the MCvar1 CIDRα2.8 domain by SEC-MALLS.

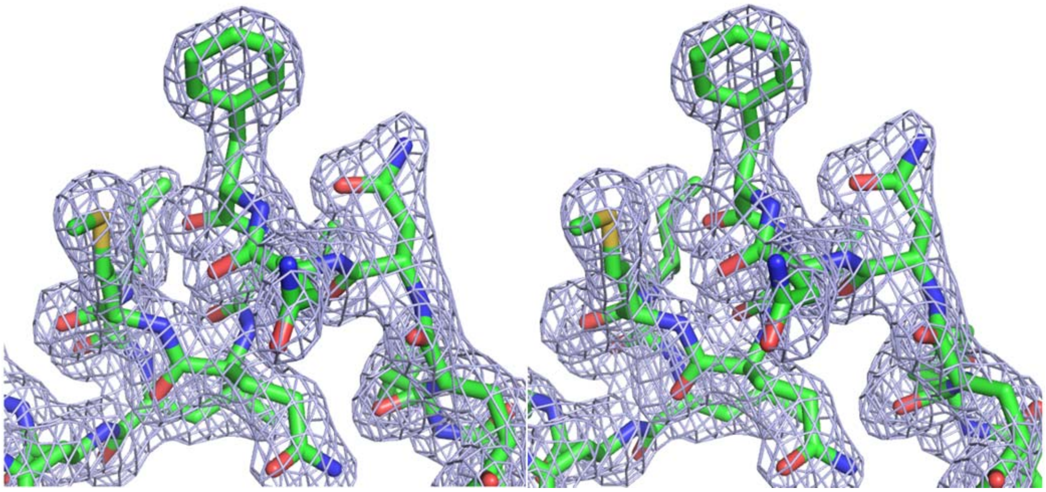

Supplementary Figure 2: Stereo image of electron density

A stereo image of the electron density for residues 148-160 of CD36. This is representative of the quality of electron density throughout the complex and contains the important residue, F153, which lies at the interface with the MC179 CDIRα2.8 domain.

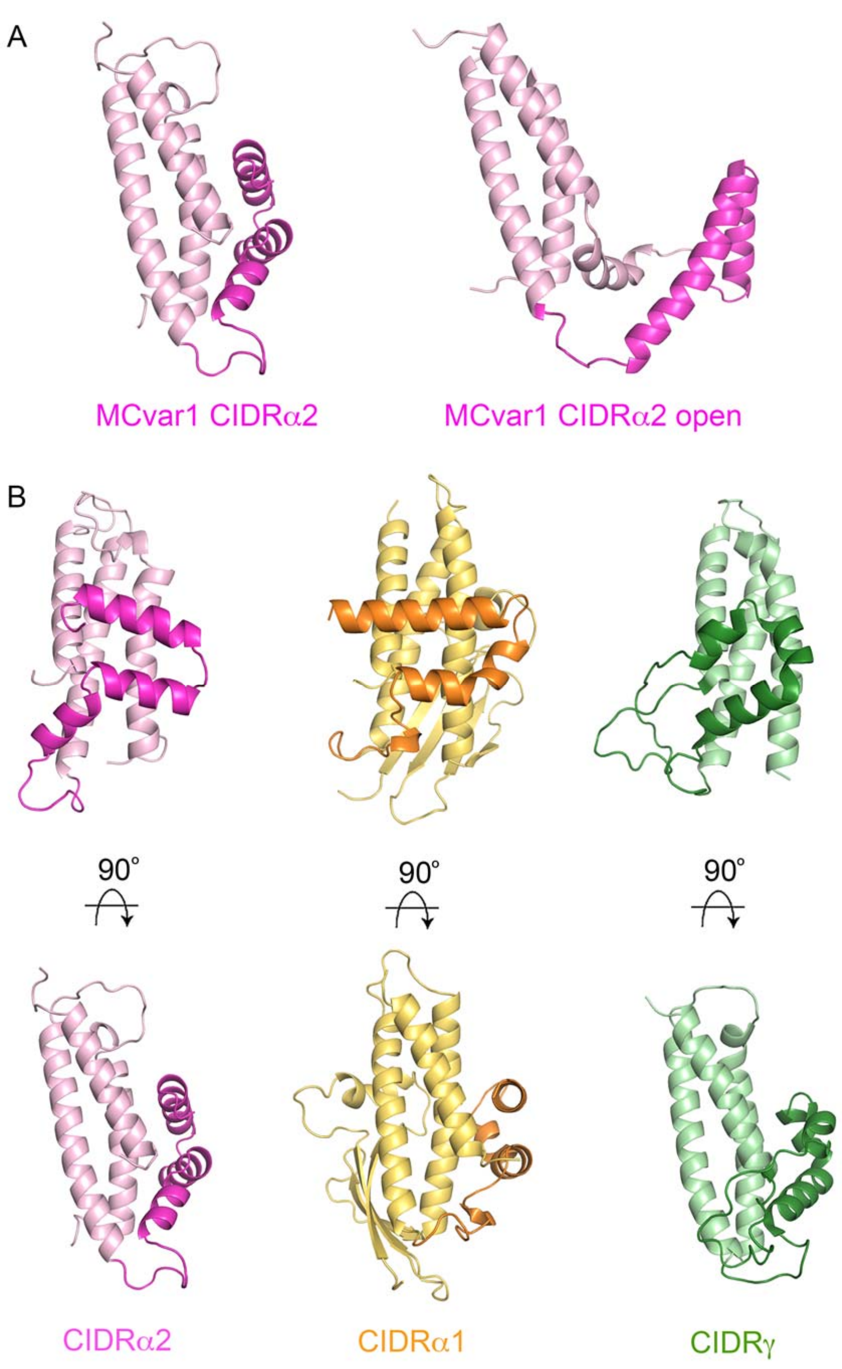

Supplementary Figure 3: Structural comparison of CIDRα domain structures

A. The structure of the MCvar1 CIDRα2.8 domain determined in this study in comparison with the previously determined 'open' conformation of the MCvar1 CIDRα domain, obtained in a pH 4.2 crystallisation condition (pdb:3C64). **B.** Comparison of the MCvar1 CIDRα2.8 structure, determined in this study with previous CIDRα1 (pdb:4V3D) and CIDRγ (pdb:2YK0) structures reveals these to adopt a compact structure with a core three α-helical bundle and the insertion linking the second and third helices packing against the rest of the domain.

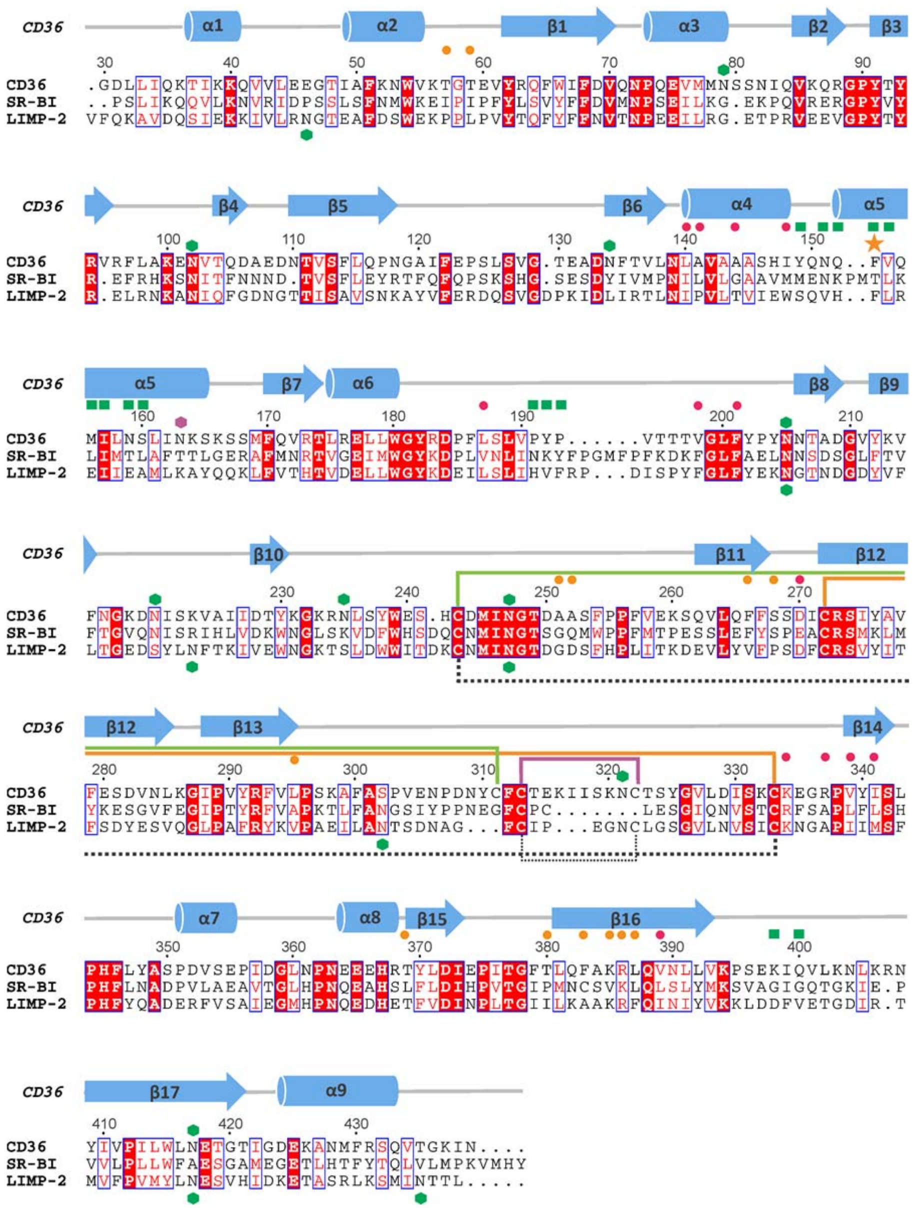

Supplementary Figure 4: Analysis of CD36 and its homologues

An alignment of the sequence of CD36 with those of its homologues SR-BI and LIMP2. Above the alignment is the secondary structure of CD36, derived from the crystal structure. Orange and pink circles show residues that interact with the two fatty acids while green squares show those that interact with the MCvar1 CIDRα2.8 domain. Orange, green and purple lines show the positions of the disulphide bonds of CD36; and dotted lines show the disulphide bonds of LIMP-2. Green hexagons show the positions of N-linked glycans on CD36 (above the sequence) and LIMP-2 (below the sequence) while the purple hexagon shows a position predicted to be glycosylated on CD36 but without a glycan.

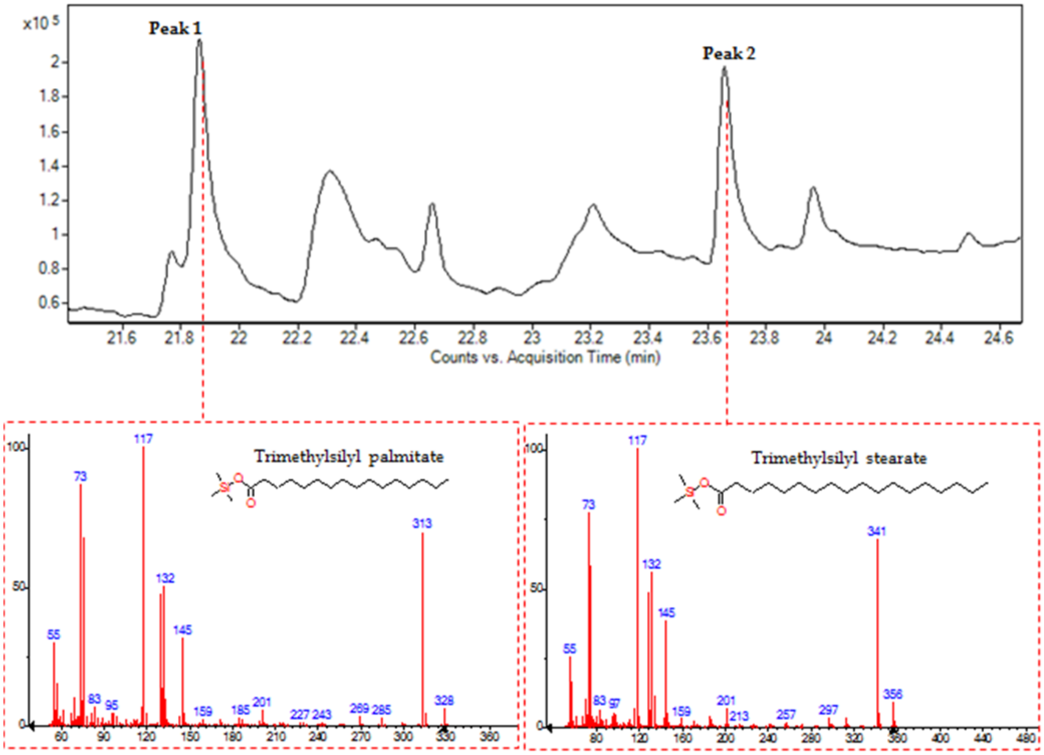

Supplementary Figure 5: Fatty acid identification by mass spectrometry

Fatty acids were chloroform extracted from the protein used for crystallisation and were converted to their corresponding trimethylsilyl esters and analysed by GC/MS. Masses were consistent with the identification of the most prevalent species as palmitic acid and stearic acid.

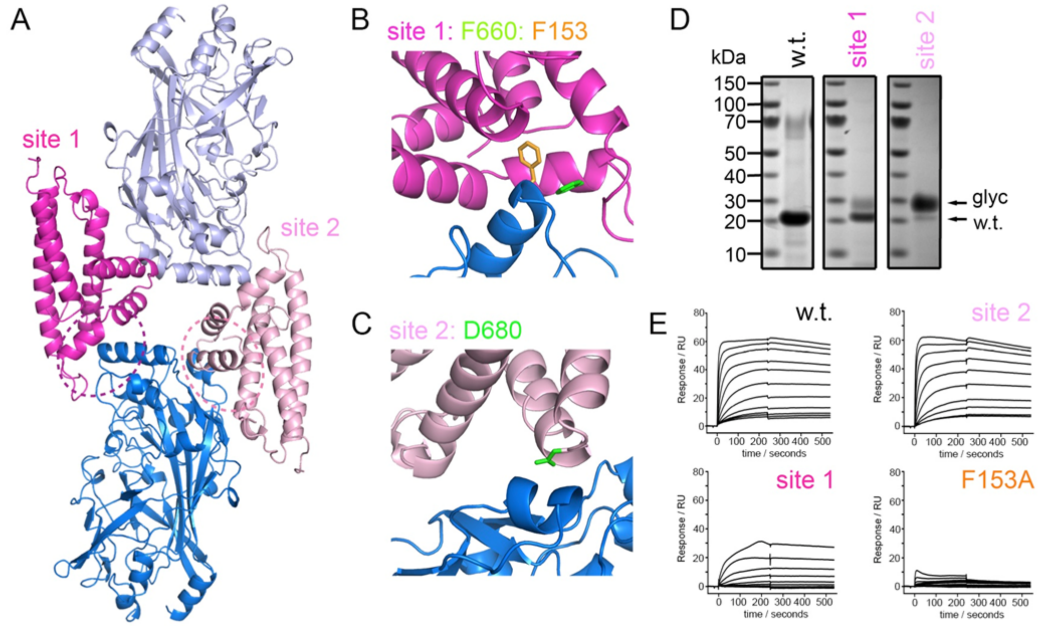

Supplementary Figure 6: Analysis of crystal packing

A. In crystals of CD36 bound to the MCvar1 CIDRα2.8 domain, two CIDRα2.8 domains (dark and light pink) interact with each CD36 (blue). **B.** A close up of site 1 with CD36 residue F153 in orange and CIDRα2.8 residue F660 in green. **C.** A close up of site 2 with CIDRα2.8 residue D680 in green. **D.** The expression of mutant forms of the CIDRα2.8 domain with N-linked glycosylation sites introduced in site 1 (F660N:Q662T) and site 2 (D680N:N682T). A small fraction of the site 1 mutant was glycosylated while the majority of the site 2 mutant was glycosylated. **E.** Surface plasmon resonance showing binding of CD36 and CIDRα mutants. Introduction of a partial site 1 glycan and the F153A mutation of CD36 both disrupted binding, while a glycan inserted in site 2 did not affect binding. This confirms that site 1 represents the interaction that occurs in solution while site 2 represents a crystal contact.

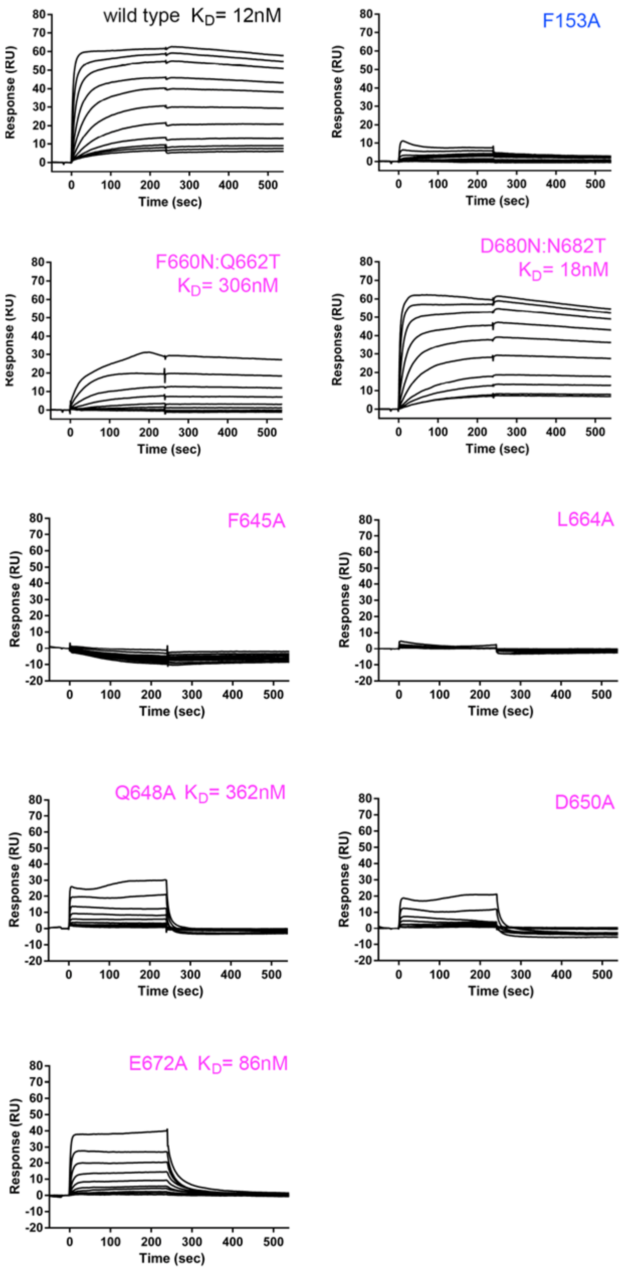

Supplementary Figure 7: Mutagenesis to assess the role of regions of the CIDRα domain in CD36 binding

Surface plasmon resonance analysis of the binding of MCvar1 CIDRα2.8 to CD36 and the effect of mutations on the interaction. The F660N:Q662T and D680N:N682T mutants introduce glycosylation sites into potential binding interfaces. The F153A mutation of CD36 and the F645A and L664 mutations of MCvar1 CIDRα2.8 disrupted the hydrophobic core of the binding interface. The Q643A, D650A and E672A mutations disrupted additional hydrogen bonds formed between CD36 and MCvar1 CIDRα2.8. Each set of binding curves shows a series of two-fold dilutions from a starting concentration of 400nM.

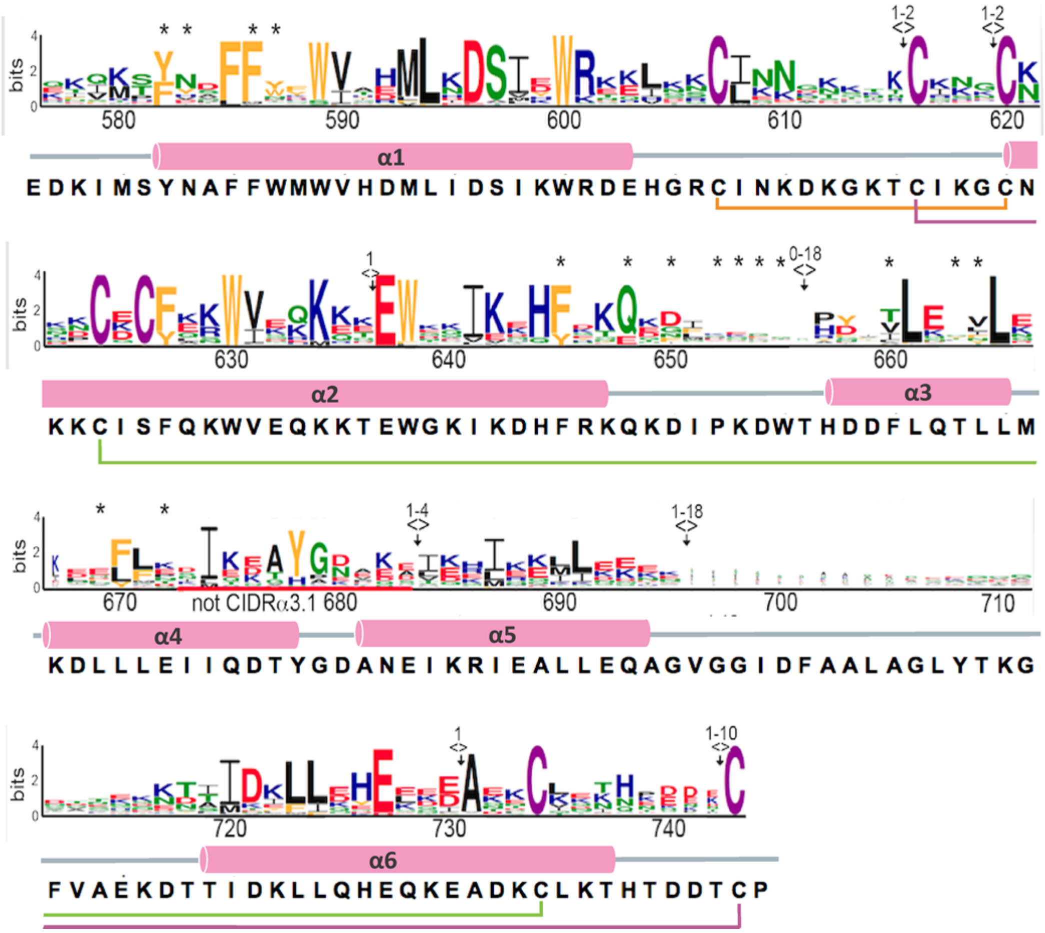

Supplementary Figure 8: Conservation across the CD36-binding CIDRα2-6 domains

Sequences of 2386 CIDRα2-6 domains were aligned and a sequence logo generated of residues equivalent to those found in the MCvar1 CIDRα2.8 domain. Deletions (><) and insertions (<>) are indicated and underneath the sequence is a representation of the secondary structure. The green, purple and orange lines show the positions of disulphide bonds and the stars above the logo denote the residues that directly interact with CD36.

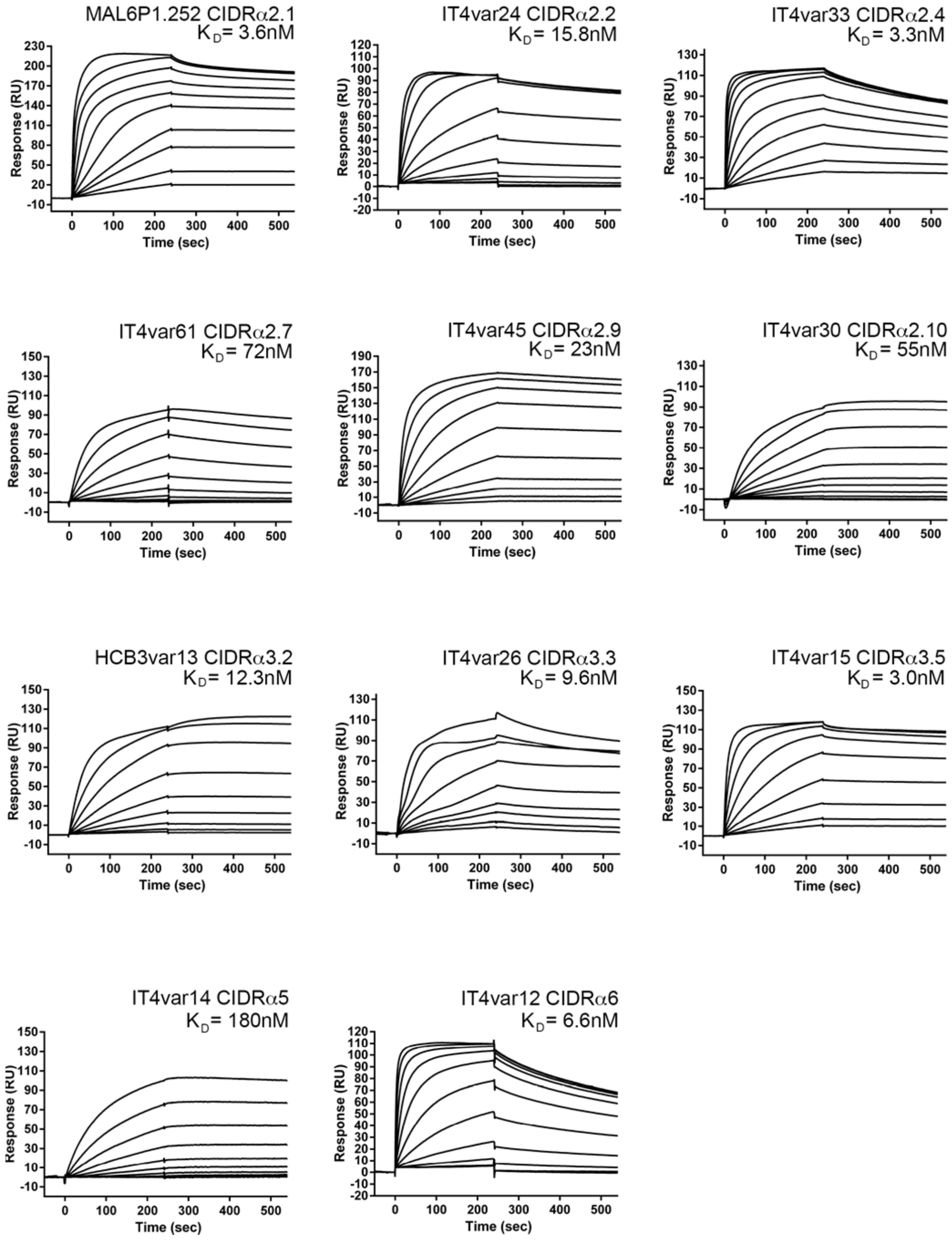

Supplementary Figure 9: Analysis of the binding of CIDRα2-6 variants to CD36

Surface plasmon analysis of the binding of different CIDRα2-6 subclasses to CD36. Each set of binding curves shows a series of two-fold dilutions from a starting concentration of 400nM.

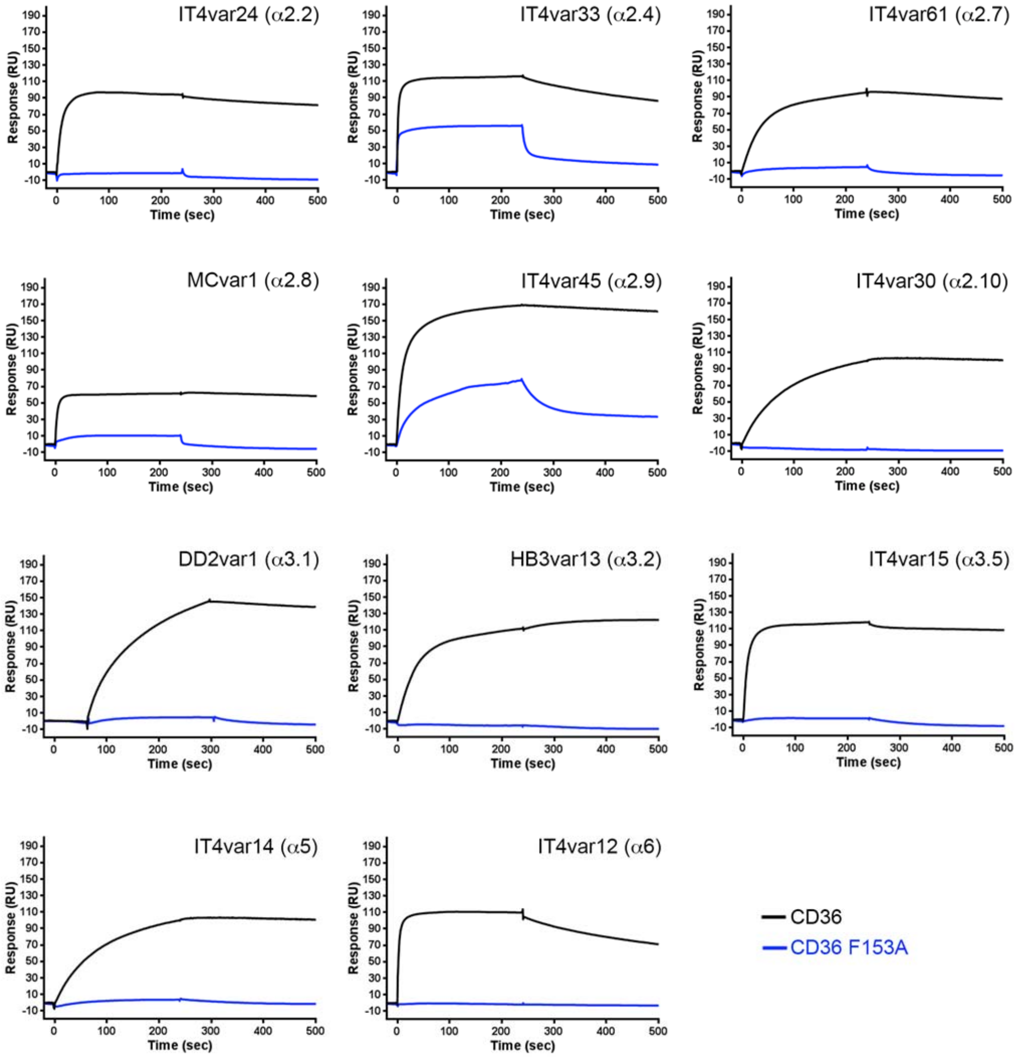

Supplementary Figure 10: The effect of the F153A mutation of CD36 on the binding of CIDRα2-6 variants

Surface plasmon analysis of the binding of equivalent concentrations of different CIDRα2-6 subclasses to CD36 (black) and the F153A mutant of CD36 (blue). In each case, the concentration of CIDRα domain was 400nM.

Supplementary Table 1: The interactions between CD36 ectodomain with CIDRα2.8

CD36		MCvar1:CIDRα2.8		
**Residue**	**Group**	**Residue**	**Group**	**Interaction**
TYR149	side chain	PRO652	side chain	Hydrophobic
ASN151	side chain NH2	GLN648	side chain CO	Hydrogen bond
ASN151	side chain NH2	ASP650	side chain	Hydrogen bond
GLN152	side chain NH2	ASN583	side chain CO	Hydrogen bond
PHE153	side chain	TRY582	side chain	Hydrophobic
PHE153	side chain	ASN583	back bone	Hydrophobic
PHE153	side chain	PHE586	side chain	Hydrophobic
PHE153	side chain	TRP587	side chain	Hydrophobic
PHE153	side chain	PHE645	side chain	Hydrophobic
PHE153	side chain	PHE660	side chain	Hydrophobic
PHE153	side chain	LEU664	side chain	Hydrophobic
VAL154	side chain	PRO652	side chain	Hydrophobic
VAL154	side chain	PHE660	side chain	Hydrophobic
MET156	side chain	TRP587	side chain	Hydrophobic
MET156	side chain	ILE664	side chain	Hydrophobic
MET156	side chain	ILE673	side chain	Hydrophobic
ILE157	side chain	TRP655	side chain	Hydrophobic
ILE157	side chain	PHE660	side chain	Hydrophobic
ILE157	side chain	THR663	side chain	Hydrophobic
ILE157	side chain	LEU664	side chain	Hydrophobic
ILE157	side chain	LEU669	side chain	Hydrophobic
ASN159	side chain NH2	ASP676	side chain	Hydrogen bond
SER160	side chain	GLU672	side chain	Hydrogen bond
PRO191	backbone O	TRP655	side chain NH	Hydrogen bond
TYR192	side chain	VAL154	side chain	Hydrophobic
TYR192	side chain	TRP655	side chain	Hydrophobic
TYR192	side chain	PRO652	side chain	Hydrophobic
PRO193	side chain	TRP655	back bone	Hydrophobic
PRO193	side chain	ASP654	back bone	Hydrophobic
LYS398	side chain NH3	ASN583	side chain CO	Hydrogen bond
GLN400	side chain NH2	ASP676	side chain	Hydrogen bond

Supplementary Table 2: Primers used in cloning

Primers for cloning CD36 and MC179var1 CIDRα2.8:

CD36 ectodomain(35-439)	
Primer 1	ctccaccggtcagaagacaattaaaaagcaag
Primer 2	gtggggtaccctattagtttatttttccagttacttgacttc
MCvar1 CIDRα2.8	
Primer 1	ctccaccggtgaggacaagattatgagctac
Primer 2	gtggggtacc tggtgggcatgtgtc

Primers for mutagenesis:

CD36_F153A	
Primer 1	catatctatcaaaatcaagccgttcaaatgatc
Primer 2	gatcatttgaacggcttgattttgatagatatg
MCvar1 CIDRα2.8_ F645A	
Primer 1	aagatcaaggaccacgccagaaagcagaaggac
Primer 2	gtccttctgctttctggcgtggtccttgatctt
MCvar1 CIDRα2.8_ Q648A	
Primer 1	gaccacttcagaaaggccaaggacatccccaag
Primer 2	cttggggatgtccttggcctttctgaagtggtc
MCvar1 CIDRα2.8_ D650A	
Primer 1	ttcagaaagcagaaggccatccccaaggactgg
Primer 2	ccagtccttggggatggccttctgctttctgaa
MCvar1 CIDRα2.8_ L664A	
Primer 1	gactttctgcagaccgccctgatgaaggacctg
Primer 2	caggtccttcatcagggcggtctgcagaaagtc
MCvar1 CIDRα2.8_ E672A	
Primer 1	aaggacctgctgctggccatcatccaggacacc
Primer 2	ggtgtcctggatgatggccagcagcaggtcctt
MCvar1 CIDRα2.8_ F660N:Q662T	
Primer 1	tggacccacgacgacaacctgaccaccctgctgatgaag
Primer 2	cttcatcagcagggtggtcaggttgtcgtcgtgggtcca
MCvar1 CIDRα2.8_ D680N:N682T	
Primer 1	caggacacctacggcaacgccaccgagatcaagcggatc
Primer 2	gatccgcttgatctcggtggcgttgccgtaggtgtcctg

Supplementary Table 3: CIDR domain sequences for recombinant proteins

> CIDRα2.8 MCvar1
EDKIMSYNAFFWMWVHDMLIDSIKWRDEHGRCINKDKGKTCIKGCNKKCICFQKWVE
QKKTEWGKIKDHFRKQKDIPKDWTHDDFLQTLLMKDLLLEIIQDTYGDANEIKRIEA
LLEQAGVGGIDFAALAGLYTKGFVAEKDTTIDKLLQHEQKEADKCLKTHTDDTCPPQ
EDRSVARS

>CIDRα6 IT4var12
PWCGLKKQADGTWKRLYENDPQCPIKPKYEPPKGEEPTEIDVLYTGKENKDIIVKLR
EFCKTDGNTGFKNEEWNCYYQVGNDKCVLENGEELGGEKKVKDYDNFLMFWVAHMLK
DSIEWRSKLSNCLKSDKKTCIKKCNDNCKCYEKWIGKKKVEWTQIKKHFDKQTDFQG
WGRYFVLETVLEGDQFFTDITKAYGDAREIVHIQEMLQKKKEQVLHEDASNMKTIID
ELLDHELKEAKQCIVNHKDNNC

>CIDRα5 IT4var14
PWCGIEEQKDGKWKRINDHSACKEEELYTPKENAKYTKINVLTSGEGHEDIAKRLKE
FCTKTQNGGGGSDDCGGNSDSSLCEPWQCYQPDQLEKVGGGEVDDKLKGAGGLCIFE
KMKGEKKVKKQKTFNNFFNFWVAHVLKDSIDWRTQLTKCLSEDKLKKCEKGCKSNCE
CFKKWIEKKEKEWIKVKDQFNKQTDFLEWKHYLVLETILENYYFENIQKAYGDLKSI
QEMKKMIKENKQNKNRTKDDEDALDVLFDHEKEEAEDCLDIHE

>CIDRα2.2 IT4var24
PLCGVNGPKGNWTDIEDKECTLVEKKKYDSNNTTNIDILTADKSQKNILQKYNKFCK
NGANDEKSATPTANGGGQIKNWQCYYDESKESGQNNNCILGKWEDFTGNEDVTSYNV
FFYNSIIEMLNDSIEWKDKLNICINNKTGKCRKVCKNPCECYKRWIEKKKTELEKIK
DHFRKQKDIGDAAQREMTLNITLNNNFLNDIKDAYPVKQQLQKIEERLKDKMQEDFI
FERTKTSIDKFLQEEEQFAEKCIEKHKC

>CIDRα2.10 IT4var30
PWCGIEEQKVDGKWKAKNNGECAKVENKIYDPKKTTTIEILTGDNEKFGIYEKYNKF
CANGKNGVTSGAPDTAPGKNGNQIVTWQCYYDKKKRSGQNNDNCVEGTWEKFTGKQT
VKSYNVFFWDWVYHMLHDSLEWRERLNSCINNAKSQNCKNNKCNRECGCFAKWVVKK
KDEWDKIKDHFNKQENIGQNVGFIEFNHYGVLEGVLDKDELLKNIKDTHADAKDIER
IDKMLQQAGVVGGGRGGENNTIIDKFLQEEERFAKDCQQKQEEC

>CIDRα2.4 IT4var33
PLCGLGMQEPPWNPKKDTDCEDRGIKTFDDRNSTPINLLVKDVTGTSIVEKLGGLCG
NGAKKNIQTWKCRFESSQNYYCVLQNDKKNTPQQEIESFNSLFWHWITEMLKDSIDW
RKEHENCINNNNTCKKGCKSKCECFEKWVKRMKEEWKQVLEVYDKQPDFKEVFTPYF
TLGYLLKEYFTKIKAPYEEVESVQEFIKEMEQIIDENSNNINATKENNSITKFLQHE
EGIATECKKTHNEEKC

>CIDRα2.9 IT4var45
PWCGVNGTKGNWTPKNDDDCKPGNEYKNYENTQIPILTGDKTKSEIVERYRKFCKNN
GKNGANGREGGVGGSENGAASNSDNATTGYCGTNNNDKDPSLCEKWTCYYKKKEKND
GKKAINFCVLQDDKVGKSQEKSMHYNSFFWDWVYHMLHDSLDWRKQLGRCINKKEET
KCIGSCNKKCECFAKWVGHKQQEWEQIKKHFLKQDDIGQETNCDPMVTLEILLDIDE
LLKNIKDTHANADDIDRIQNMLQQAGFDGGVAALVGRCTEGFVAEKDTTIDKFLEKE
LQEAEKCLETHKEKC

>CIDRα2.7 IT4var61
PWCGVKRNGGGWKAKSDGECAKEKKTYKKKNITEIPVLTPDKEKHNILQKYENFCKN
SDGNNGDQIKNWQCYYEEKDESDNDGDSNICVLQNENIGKKEEKSMPYHPFFWKWVT
EMLIDSMYWRKELKRCINKETKACKNGCKNNCDCYKRWVEEKKKEWGQIKTHFNTQE

DMREDIGENTDPGIILAALLNIEDLFENIKDTYGDVKEIKDINQMLEKENEENEGTA
GADSKKKNTIDLMINHEQKDAQKCVTNNPDKDC

>CIDRα3.2 HB3var13
PLCGVKKEKGTWVRKDSMNDCPRIKLYKPINDKVGTPINFLYSGDGQTEIAEKLKKF
CRTENGSDGSSSARANGASGDKNGGSGSQELYQYWKCYQIGDLQKVREGEDDEDDGQ
YDQEVENAGGLCILQKTNGKENVNKQKTSHEIQKTFNPFFYYWVVHMLKDSIHWRTE
KIKSCINNTNELKACKNNKKCNSDCDCFKRWVKQKGKEWGQIKVHFKTQDIRGKVVN
GNTVVSFFLDHDELLEGVLDKGLLLESLQEAYGNAEDIKHIKKLLQETDVVGGGEHK
TTIDFLLKEELNEAEECLKKQKDNEC

>CIDRα3.5 IT4var15
PGCGVELIGNEWKEKNKGECKGGKRYNIPKGTKHNVIPVLSFGDEHKEIIEKIEQFC
AESNSDSSKLTEQWKCYYGDKEYEVCTLENRNKSEEDPEEIQKTFHNFFYFWIRHLL
NDSIEWRDKINNCIEKAKEGKCKNECKTDCGCFQRWIGKKKEEWGEIKKHFKTQDGF
SIFGNNYDFVLENVLNIDELFQDITEAYGNSQKIQGIKDTLAKKKTQAADDATEQKN
TIDLLFEYDSEEAEKCKKIQEEC

>CIDRα2.1 MAL6P1.252
PWCGVKPGGPPWKDNDIDSCGKKEISFSDKDTTDISILSTDRAKKNILQKLENFCRD
TEHINHDIWKCHYENTDNDNCILQNENTGSEKQKIMPFDAFFFLWLTQMLDDSIEWR
KKLKTCINNEKPTNCIRGCKKPCECFERWVEQKEEEWISIEKHFDKQRDISEEERYI
TLEYILNEFFMDKIEKAYGIEKSKELKEKLKSNKGHGIIRDTEHSQDAIKILLEHEL
EDAKKCTETHNDEKC

>CIDRα3.1 DD2var01
PYCGVKKVNNGGSSNEWEEKNNGKCKSGKLYEPKPDKEGTTITILKSGKGHDDIEEK
LNKFCDEKNGDTINSGGSGTGGSGGGNSGRQELYEEWKCYKGEDVVKVGHDEDDEED
YENVKNAGGLCILKNQKKNKEEGGNTSEKEPDEIQKTFNPFFYYWVAHMLKDSIHWK
KKLQRCLQNGNRIKCGNNKCNNDCECFKRWITQKKDEWGKIVQHFKTQNIKGRGGSD
NTAELIPFDHDYVLQYNLQEEFLKGDSEDASEEKSENSLDAEEAEELKHLREIIESE
DNNQEASVGGGVTEQKNIMDKLLNYEKDEADLCLEIH
